# Advanced Online Survival Analysis Tool for Predictive Modelling in Clinical Data Science

**DOI:** 10.1371/journal.pone.0161135

**Published:** 2016-08-17

**Authors:** Julio Montes-Torres, José Luis Subirats, Nuria Ribelles, Daniel Urda, Leonardo Franco, Emilio Alba, José Manuel Jerez

**Affiliations:** 1 Computer Science Department, Malaga University, Malaga, Spain; 2 Yachay Tech University, Urcuqui (Imbabura), Ecuador; 3 Virgen de la Victoria University Hospital, Malaga, Spain; 4 Malaga Biomedical Research Institute (IBIMA), Malaga, Spain; Texas A&M University, UNITED STATES

## Abstract

One of the prevailing applications of machine learning is the use of predictive modelling in clinical survival analysis. In this work, we present our view of the current situation of computer tools for survival analysis, stressing the need of transferring the latest results in the field of machine learning to biomedical researchers. We propose a web based software for survival analysis called OSA (Online Survival Analysis), which has been developed as an open access and user friendly option to obtain discrete time, predictive survival models at individual level using machine learning techniques, and to perform standard survival analysis. OSA employs an Artificial Neural Network (ANN) based method to produce the predictive survival models. Additionally, the software can easily generate survival and hazard curves with multiple options to personalise the plots, obtain contingency tables from the uploaded data to perform different tests, and fit a Cox regression model from a number of predictor variables. In the Materials and Methods section, we depict the general architecture of the application and introduce the mathematical background of each of the implemented methods. The study concludes with examples of use showing the results obtained with public datasets.

## Introduction

Over the years, a number of works [1] [2] have shown the advantages of using the latest advances in machine learning to assist clinicians in determining the clinical outcome of patients after surgery. At present, there is a wide range of software solutions aimed to provide researchers with computer tools to perform classical statistical analysis and to implement those new machine learning techniques in their works. Many of them are commercial applications such as SPSS, Stata or SAS, which enjoy considerable popularity among researchers in the field of survival analysis [3] [4]. However, this kind of software has two significant disadvantages for many users: firstly, they are proprietary applications with major restrictions on its use; secondly, new methods published in scientific literature are not incorporated and updated in a short period of time. Clinicians may find a free alternative in open source statistical computing languages such as R (R Development Core Team, unpublished data), which is rapidly growing into the gold standard in clinical research and Bioinformatics. Still, R is an interpreted programming language with a hard to learn command line syntax, and full of options of no use for biomedical researchers who are only interested in survival analysis.

This situation has motivated the birth of many applications intended to ease the work of researchers in this field, such as CanSurv [5] and PODSE [6]. CanSurv is a Windows program developed to generate graphs representing standard survival models for population based data. On the other hand, PODSE is a MATLAB based tool for parameter optimisation in discrete time survival analysis. Some other applications are web based tools, like PROGgene [7], which includes more than 130 cancer datasets and is intended to help researches to find prognostic mRNA biomarkers performing usual survival analysis techniques. KMPlot [8] is another online software for biomarker assessment that also uses its own gene expression data. Finally, there are web applications which let the users analyse their own datasets, like OASIS [9], focused in classical survival models as well.

Nevertheless, none of the mentioned programs is transferring the results of machine learning research into a practical clinical application. Furthermore, they generally lack some features that we consider essential for scientific software that is not aimed at computer specialists: i) A wide range of tools updated with the latest results published in scientific literature, ii) advanced computational methods, like ANN-based algorithms, which are freed from the proportional and linearity constrains of classical models, having the potential to obtain a more accurate clinical outcome for individual patients, and iii) a clean and straightforward user interface which, at the same time, provides a great deal of options.

In this paper we propose the design and development of OSA (Online Survival Analysis), a system which fully meet all the requirements previously mentioned, providing an easy tool to obtain personalised survival curves from ANN-based models, and to carry out traditional survival analysis. The rest of the paper is organised as follow: Materials and Methods describes the architecture of the application, the web site structure and the mathematical foundations of the methods; Results and Discussion shows some results obtained with public datasets; Conclusions summarises our work and mentions future improvements. OSA is free and can be found at: http://www.icb.uma.es/Survival.

## Materials and Methods

### Architecture

OSA consists of the following elements:

1. An IIS web server running an ASP.NET MVC 4 application, which takes advantage of the latest ASP.NET, JavaScript and HTML5 technologies to provide an easy to use and feature rich user interface.
2. An SQL Server database which safely stores all the uploaded information using the 256 bit AES encryption to protect sensitive data such as the user’s password.
3. A computational cluster with 27 nodes that executes the tasks by means of the R environment.

**Fig 1:**
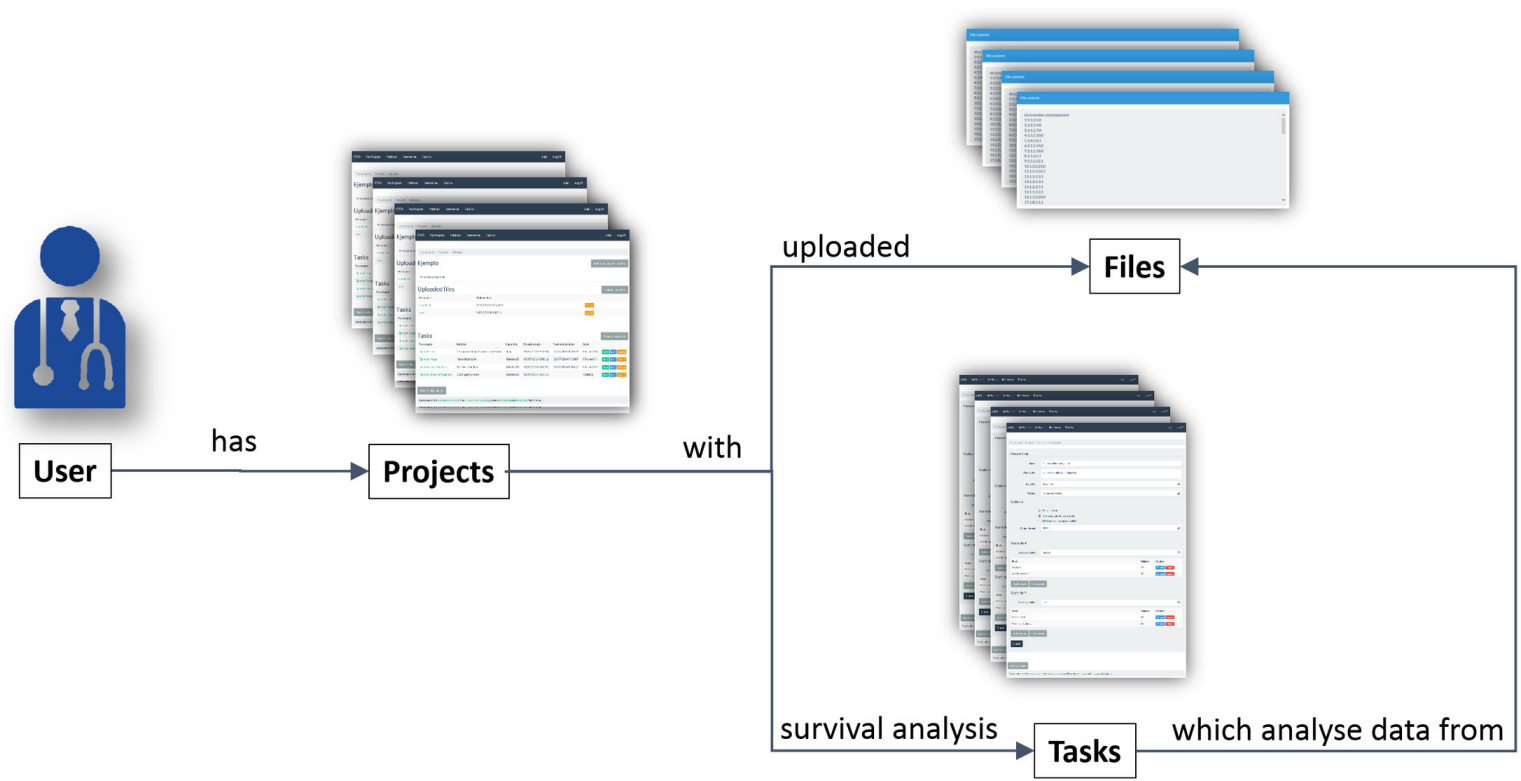
Simplified application structure diagram. This figure shows the main entities involved in the application workflow and their relationships.

Fig 1 shows a simplified diagram of the application structure. The user can create projects which contain uploaded files with datasets and tasks to analyse the data. An IIS web server accesses the application database with the ADO.NET Entity Framework. It also connects to a web service that processes all the task execution requests. The web service selects a free node, constituted by a quad core x64 PC with 4 Gigabytes of main memory running Linux. Next, the task is executed in that node using the R environment and the result is collected by the web service, which sends it to the main application.

### Website structure

**Fig 2:**
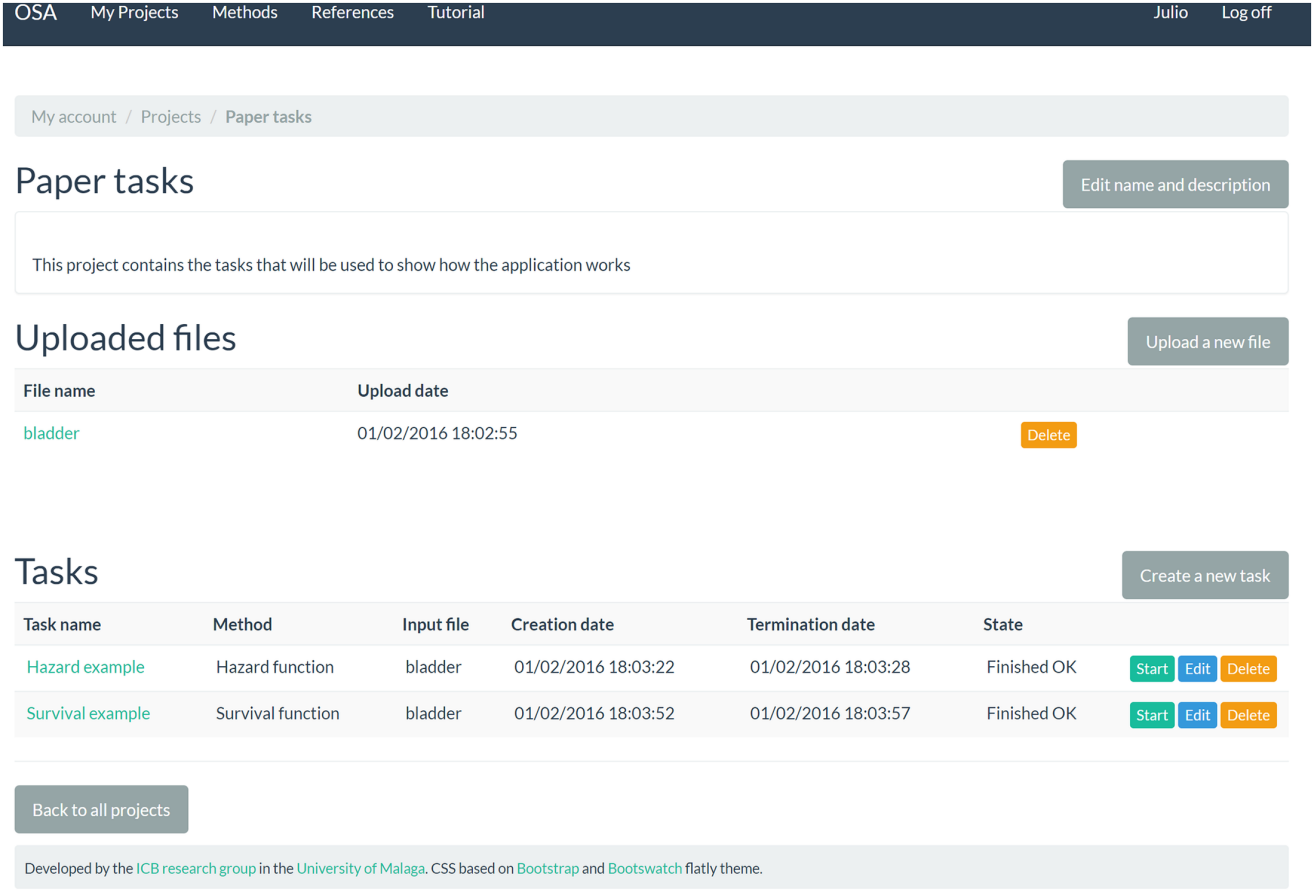
Screenshot of a project page. Here, the user can upload datasets and create tasks.

Every web page in the application features a top menu bar as in Fig 2, with the next five links:

1. My Projects. When logged on, it leads to the user’s project list; otherwise the log on formulary will be shown to the user. Each project plays the role of a container where files with survival data and task are stored.
2. Methods. This link provides access to the method description web page, in which we include all the information regarding the mathematical background of the available statistical procedures.
3. References. Following this link, every visitor can refer to the supporting references of this work. These are presented in an appropriately formatted list.
4. Tutorial. It points to a concise explanation of the usage of this web application with practical examples.
5. Log on. This is the link to the log on formulary, where the user has to enter the name and password in order to access to the personal area. The formulary also includes a link to the register page for those who need to create a new account.

After signing up, an empty project list and a button to create new projects will appear. Once the project has been created, it is possible to include CSV files with survival data to perform statistical analysis. The file upload process requires the user to provide some basic information about the data, like the names of the columns with the follow-up time and the censorship status. When there is at least one CSV file in the project, the user can select one of the four traditional statistical methods or the ANN-based method provided by the application by creating a new task. Finally, after clicking on the start button to execute the task, the result can be obtained in the form of a table or a graph which can be downloaded in various formats (including PNG and PDF).

### Methods

The methods provided by OSA to perform statistical analysis on the uploaded data can be classified in three groups: The basic, classical survival analysis methods (which includes the survival function and the hazard curve), the Cox proportional hazards regression model and the ANN-based predictive model.

#### Kaplan-Meier survival curve

The first basic method is the survival curve, which plots a Kaplan-Meier [10] estimate of the survival function. The method is based on the R packages survival (Therneau T, Lumley T, unpublished data) and epitools (Aragon T, unpublished data). Let *t*_*i*_ be an observed event time, where *i* = 0, 1, 2, …*D*. Let *m*_*i*_ be the number of subjects with an observed event time *t*_*i*_, and *n*_*i*_ the number of subjects at risk before *t*_*i*_. Then, the Kaplan-Meier estimate, $\hat{S}\left(t\right)$, is given by

$$\hat{S}\left(t\right)=\left\{\begin{array}{cc} 1 & \text{if}\;t<t_{1}\\ \prod_{t_{i}\leq t}\left(1-\frac{m_{i}}{n_{i}}\right) & \text{otherwise} \end{array}\right.$$ (1)

The survival curve method includes a number of options, such as showing censored data, plotting the confidence interval at 95 percent, generating the plot using black and white graphics, showing the median follow-up time for one curve or including a table with the number of patients at risk. The user can also compare different survival curves in the same plot to establish whether there is a statistically significant relationship between them. The available tests to perform the comparison are Log-Rank [11], Peto-Peto [12] and Tarone-Ware [13]. Assuming the existence of two curves, X and Y, the comparison estimate would be

$$\frac{{\left[\sum_{i=1}^{p}w_{i}\left(m_{Xi}-e_{Xi}\right)\right]}^{2}}{\sum_{i=1}^{p}w_{i}Var\left(m_{Xi}-e_{Xi}\right)}\approx \chi^{2},$$ (2)

where *p* is the total number of failure times, *m*_*Xi*_ is the number of observed events at a time *t*_*i*_ for group X, *e*_*Xi*_ is the number of expected events at *t*_*i*_ and *w*_*i*_ is the weight employed by the test at *t*_*i*_. The number of expected events, *e*_*Xi*_, is computed as follows

$$e_{Xi}=\frac{n_{Xi}m_{i}}{n_{i}},$$ (3)

while the variance in Eq 2 is given by

$$Var\left(m_{Xi}-e_{Xi}\right)=\sum_{i=1}^{p}\frac{n_{Xi}n_{Yi}\left(m_{Xi}+m_{Yi}\right)\left(n_{Xi}+n_{Yi}-m_{Xi}-m_{Yi}\right)}{{\left(n_{Xi}+n_{Yi}\right)}^{2}\left(n_{Xi}+n_{Yi}-1\right)},$$ (4)

where *n*_*Xi*_ and *n*_*Yi*_ are the number of subjects at risk before *t*_*i*_ for group X and Y, respectively, and *m*_*Yi*_ is the number of subjects in group Y with an observed event time *t*_*i*_. It can be proven that all the calculations are equivalent exchanging X for Y. The tests can be generalised to compare more than two groups, in which case the comparison estimate is approximately *χ*^2^ distributed with *k* − 1 degrees of freedom, where *k* is the number of groups. For each curve, the result shows the median survival time, the number of events that have taken place and the total number of patients. It also prints the computed P value for the test, followed by the odds ratio when comparing exactly two curves. The default position of every label and legend can be selected within the plot area.

#### Hazard function estimation

The second basic method is the hazard function, which is based on the R packages muhaz (Hess K, Gentleman R, unpublished data) and survival (Therneau T, Lumley T, unpublished data). It computes a kernel smoothed hazard function from right censored data using a fixed bandwidth kernel smoothed estimator [14] [15]:

$$\hat{h}\left(t\right)=\frac{1}{b}\sum_{i=1}^{p}K\left(\frac{t-t_{i}}{b}\right)\Delta \tilde{H}\left(t_{i}\right),$$ (5)

where *b* is the bandwidth distance of *t*, *K*() is the Epanechnikov kernel function

$$K\left(x\right)=\frac{3}{4}\left(1-x^{2}\right),-1\leq x\leq 1,$$ (6)

which is appropriately replaced by the corresponding asymmetric kernels of Gasser and Müller [16] for *t* < *b* and for *t*_*D*_ − *b* ≤ *t* ≤ *t*_*D*_, and $\tilde{H}\left(t_{i}\right)$ is the Nelson-Aalen estimator [17] [18]. The user can set the bandwidth of the kernel as well as other parameters, like showing the histogram of the function or the confidence interval at 95 percent. There are options for generating black and white results and for including a table of patients like in the previously discussed method. Another common characteristic of the first two methods is the possibility of doing visual comparison between various curves. Thus, two or more hazard functions can be shown in the same plot, including for each one the maximum hazard ratio and the time it has been reached. The user can also change the original position of every information printed over the plot area.

#### Cox proportional hazards model

The fourth available method, based on the R packages survival and MASS [19], is the Cox’s proportional-hazards regression [20]. It uses the variables the user selects from the uploaded data to fit a proportional-hazards regression model of the form

$$h\left(t,X\right)=h_{0}\left(t\right)e^{\sum_{i=1}^{q}\beta_{i}X_{i}},X=\left(X_{1},X_{2},\ldots ,X_{q}\right),$$ (7)

where *h*_0_(*t*) is the non-parametric baseline hazard, *X* is the vector of time-independent predictor variables *X*_*i*_ and *β*_*i*_ are the initially unknown regression coefficients. The available methods for tie handling are Breslow [21], Efron [22] and Exact, while the mode of stepwise variable search can be selected from a number of options, namely Backward, Forward and Both. For each variable or strata included in the model, the method computes its regression coefficient, the confidence interval of the coefficient, the number *e* to the power of the coefficient and the P value of the performed Wald test. The result is shown in the form of a table, followed by the chi-square goodness of fit test and the proportional hazards assumption test results [23].

The user can also study the correlation between two variables by contingency table analysis. This method uses the R package gmodels (Warnes GR, Bolker B, Lumley T, Johnson RC, unpublished data) for some calculations. The application allows selecting and modifying the variables from which a contingency table with marginal values will be computed. There are three tests that can be carried out using the information in the table. These are the Pearson’s chi-square test of independence, the Fisher’s exact test for small samples and the McNemar’s test [24]. In addition, the user can select whether the proportional values in the table will be represented by percentages, as in SPSS, or the way SAS depict them, on a scale from 0 to 1.

#### ANN-based method for survival analysis

Finally, the ANN-based method for predictive modeling, based on the Mani approach [25], fits a single-hidden-layer neural network with one input for each predictor variable, and as many outputs as groups are selected by the user to split the maximum follow-up time. The output represents the hazard rate for each interval of the follow-up time, which is computed by the formula

$$\hat{h}\left(t\right)=\left\{\begin{array}{cc} 0 & \text{if}\;1\leq t\leq T_{surv}\\ 1 & \text{if}\;C=1\;\text{and}\;T_{surv}<t\leq T\\ \frac{m_{t}}{n_{t}} & \text{if}\;C=0\;\text{and}\;T_{surv}<t\leq T \end{array}\right.$$ (8)

where *T* represents the maximum value of the follow-up time, *T*_*surv*_ is the subject survival time, and *C* indicates whether the subject is censored (*C* = 0) or not (*C* = 1). For uncensored observations, the hazard is zero until the interval of the time of death and 1 thereafter. For censored observations, the hazard is zero until the interval of censoring time and then is calculated by the number of subjects with an observed death at instant *t*, or *m*_*t*_, divided by the number of subjects at risk at *t*, *n*_*t*_.

To fit the neural network, the application firstly preprocess the input data to determine the size of each the *n* intervals of the follow-up time. In order to increase the accuracy of the network, the same number of events are grouped in each interval, which can make them of different lengths. Afterwards, the *n* hazard rate outputs are calculated for every patient in the study with Eq 8.

The resulting model is obtained using a 10-fold cross validation process. In this manner, the preprocessed input is split in every iteration into a test set, a validation set and 8 training sets, each one with the same number of subjects. This validation procedure is repeated generating models with the R package nnet (Ripley B, unpublished data), with a different number of hidden-layer neurons. The network with the highest accuracy is presented as the final result. The accuracy of the model is calculated by

$$ACC=\frac{\sum_{i=1}^{N}\frac{G-|T_{surv}-T_{surv}^{\prime }|}{G}}{N}$$ (9)

where *N* is the number of observations, *G* is the number of groups or intervals of the follow-up time, *T*_*surv*_ is the actual survival time of the observation and $T_{surv}^{\prime }$ is the estimated survival time for the observation.

While this network directly estimates the hazard rate of an individual subject for each follow-up time interval, it can be easy turned into a Kaplan-Meier estimator using the formula

$$\hat{S}\left(t\right)=\prod_{i|t_{i}<t}\left(1-\hat{h}\left(t\right)\right)$$ (10)

## Results and Discussion

In order to illustrate how the application works, we will use a subset of the bladder cancer dataset present in the R package survival, consisting of the 85 observations of the first recurrence of bladder cancer for each patient [26] [27]. The variables included in the dataset are the received treatment (placebo or ThioTEPA), the number of tumours and the size of the largest tumour.

**Fig 3:**
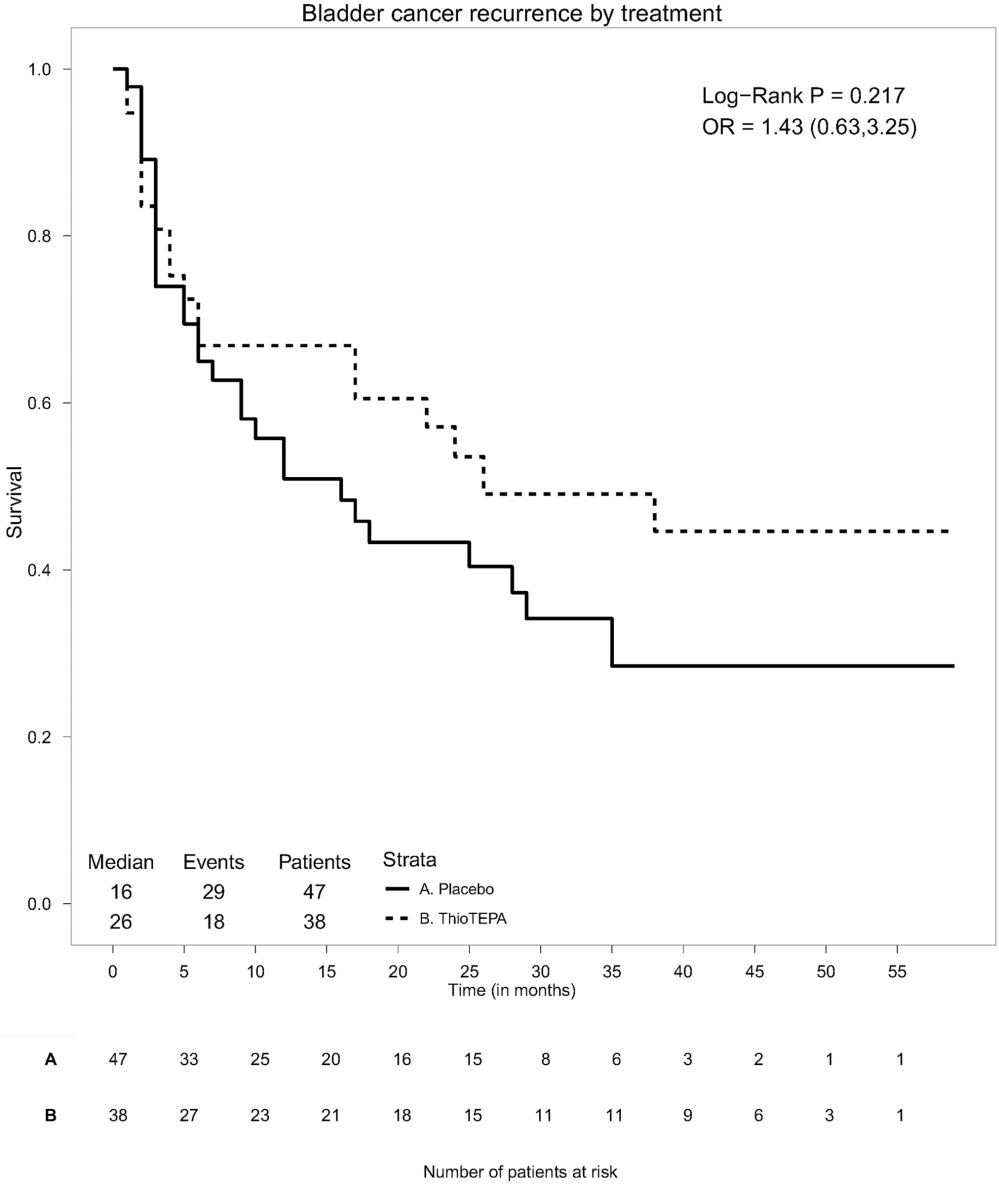
Comparison of the survival curves of two groups of patients. The plot is followed by the number of patients at risk for each group.

**Fig 4:**
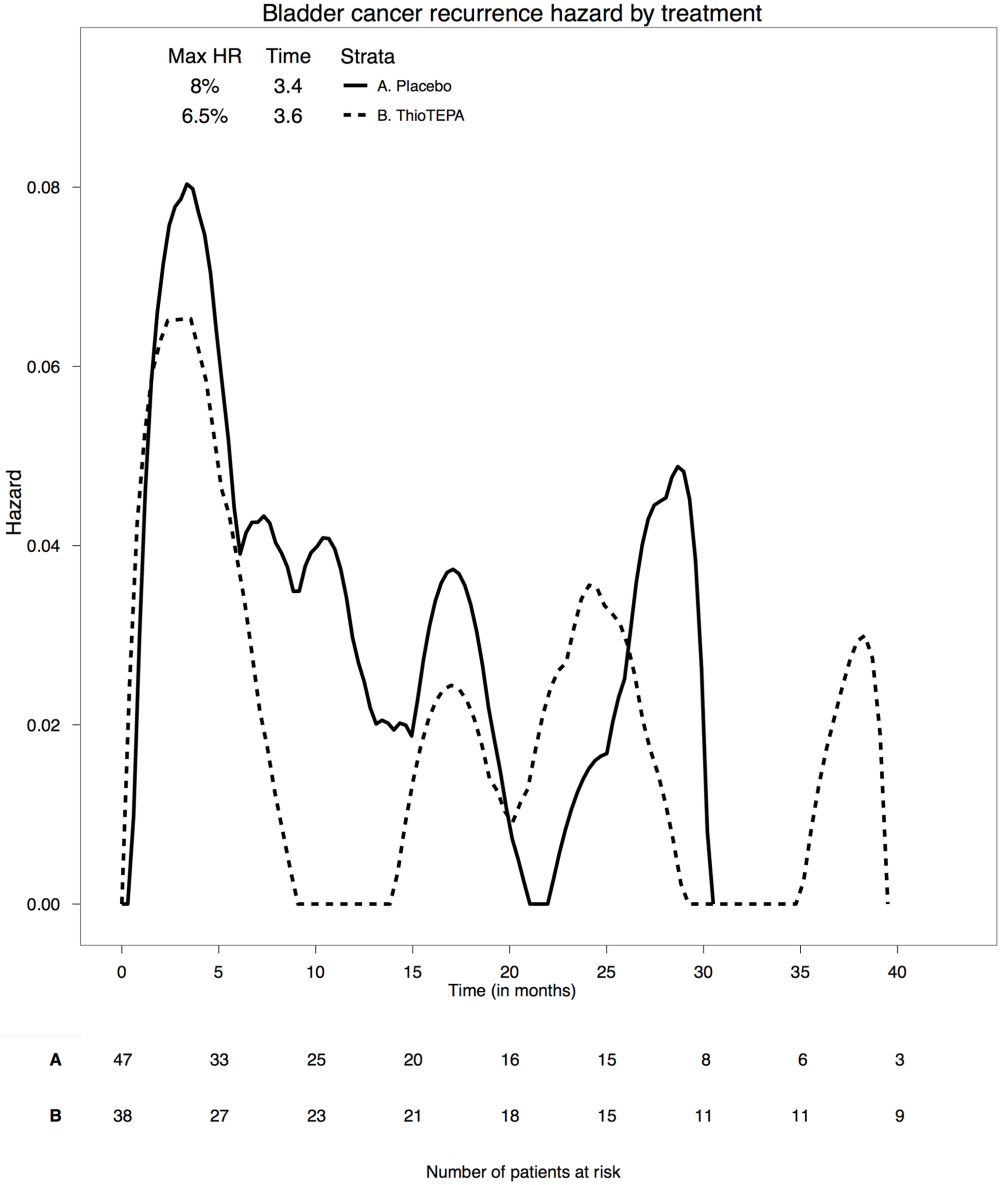
Comparison of two hazard functions. The graph is followed by the number of patients at risk.

Fig 3 shows the survival curves stratified for the two-sample treatment. In the task creation form, we set the x-axis interval to 5 months, and ticked the checkboxes to show the table with the patients at risk and to use black and white graphics. We also selected the option to compare survival distributions by the Log-Rank test and used the received treatment as the study variable. Fig 4 shows the hazard plots, obtained by using the same parameter settings as done for the survival curve. The specific options for hazard curves are the bandwidths for the kernel smoothed function. The integer bandwidth values used in this example were 3 for both the Placebo and ThioTEPA.

**Fig 5:**
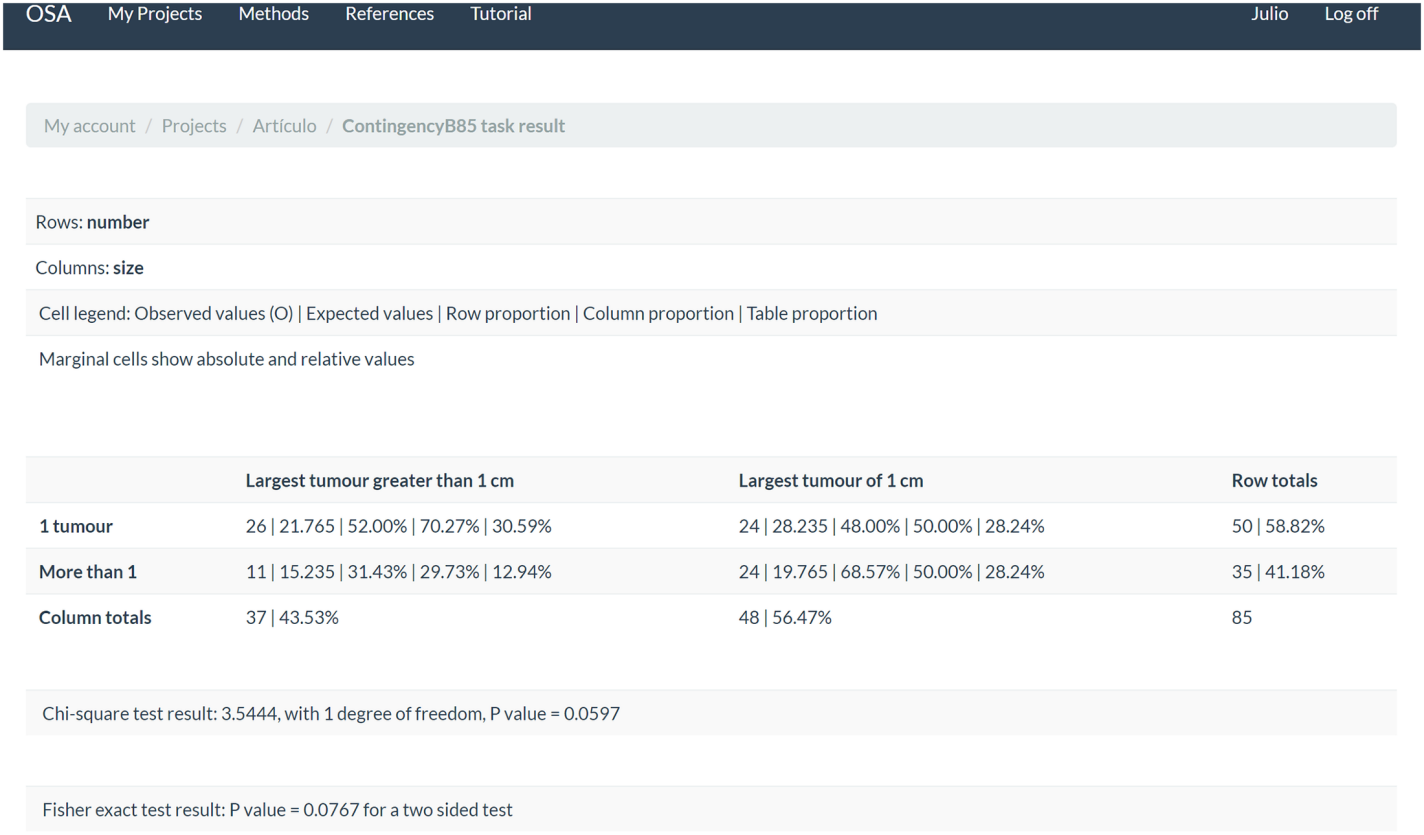
The contingency table generated by the application as it is shown on the web.

The contingency table was created to compare the relationship between the number of tumours and the size of the largest tumour, with those variables having 8 and 7 categories, respectively. For each variable, we grouped all categories but the first and renamed them taking advantage of the web formulary options. Thus, the number of tumours becomes a two-stratum variable, with categories “1 tumour” and “More than 1”, and the size of the largest tumour gets reduced to the categories “Largest tumour of 1 cm” and “Largest tumour greater than 1 cm”. Fig 5 shows the contingency table followed by the computed Pearson’s chi-square test and the Fisher exact test.

**Fig 6:**
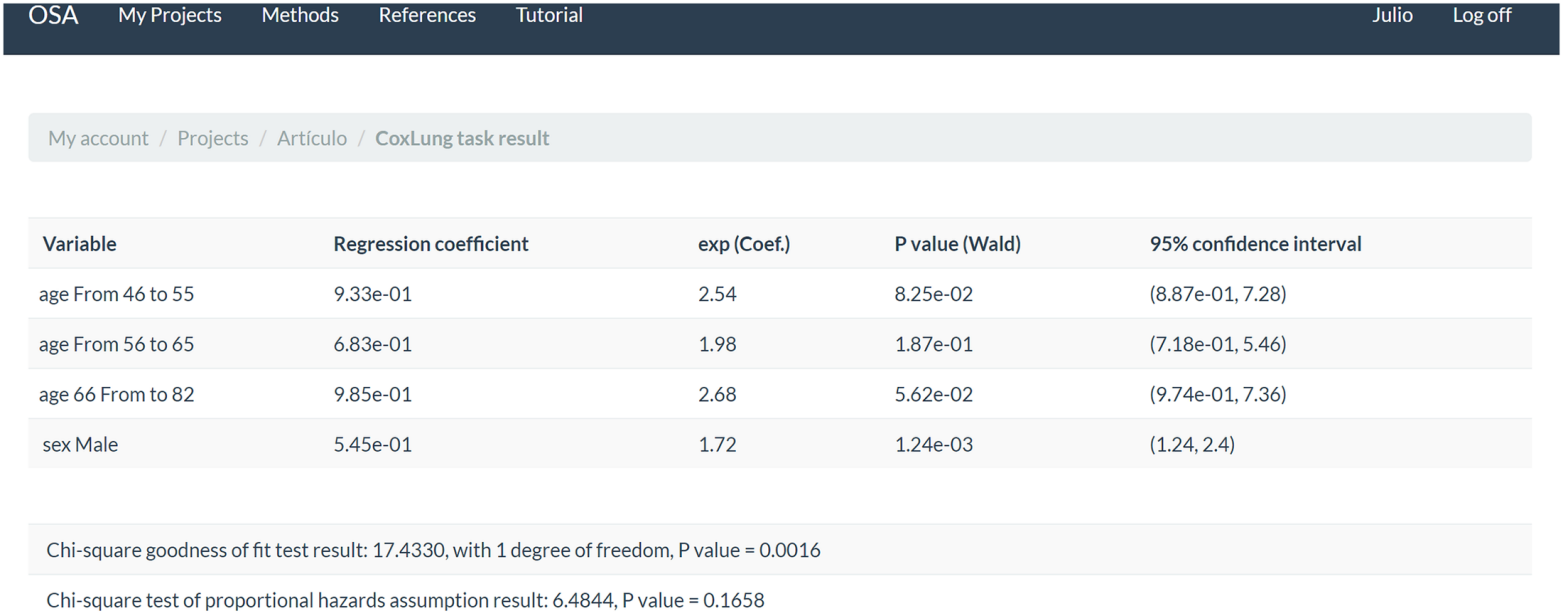
The Cox regression result as it is generated by the application.

We used a different dataset for the Cox proportional-hazards regression model. In this case, we chose the lung cancer dataset from package survival [28]. It provides variables such as the age of the patient and the sex, which were selected for this study. We created four strata for the age (ranges 39 to 45, 45 to 55, 56 to 65 and 66 to 82). Fig 6 shows the result after using the method Efron for tie handling and the backward stepwise variable search.

**Fig 7:**
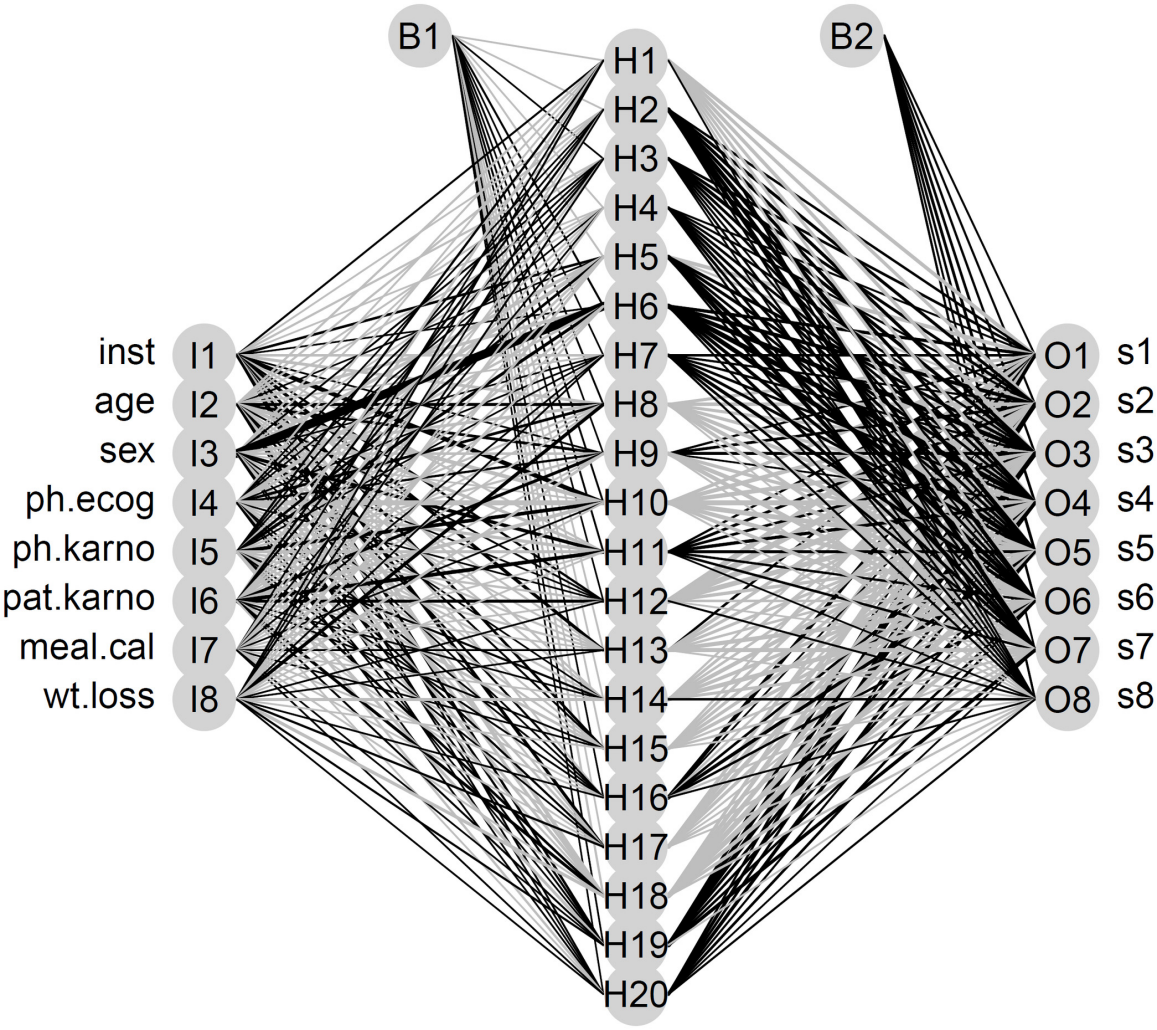
Artificial Neural Network. ANN fitted by the application, with a hidden layer of 20 neurons. The 8 inputs are the values of the lung cancer dataset variables, while the 8 outputs are the values of survival in each corresponding stratum of the follow-up time.

**Fig 8:**
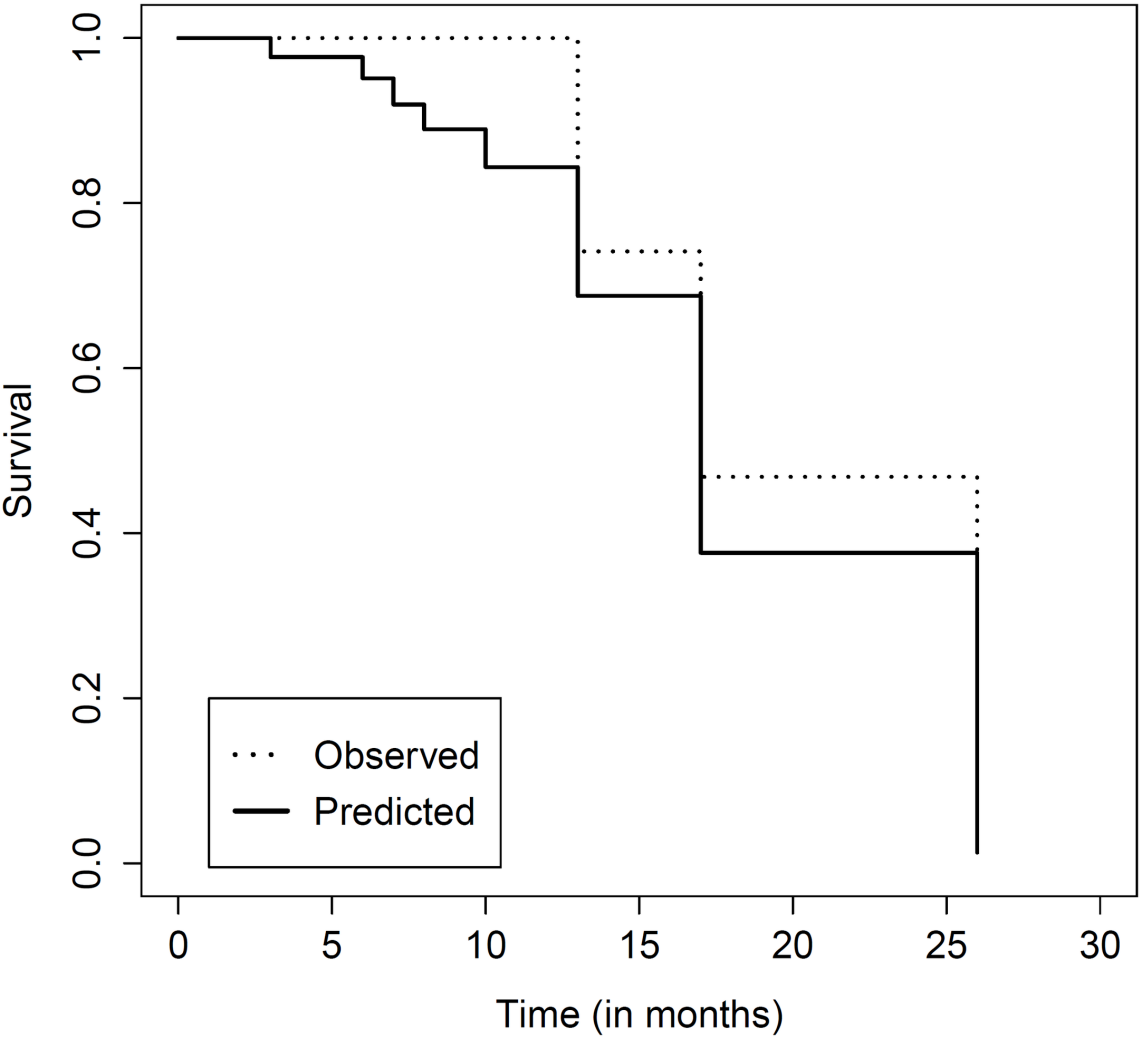
Predictive modelling. Comparison between the actual discrete time survival curve of a patient of lung cancer (dotted) and the predicted one generated by the fitted ANN-based model (solid).

Finally, we used the lung cancer dataset with the ANN-based predictor model. We stratified the age the same way we did with the previous model and discretised the follow-up time in 8 groups. Fig 7 depicts the resulting neural network which estimates the survival rate for each one of the calculated classes of the follow-up time. Fig 8 compares the actual survival curve of a specific censored patient from the dataset with the predicted one by the model. The Kaplan-Meier estimate obtained from the observed times (dotted curve) remains constant with the value of 1 until the censoring takes place. That is the reason why the first 6 stratum of the discretised follow-up time (shorter than the latter ones, as the majority of events take place at the beginning of the study) show visually significant differences between the actual (dotted) and the predicted curve (the solid one). However, after censoring, both curves develop almost identically.

## Conclusions

OSA is an easy to use and learn graphical front end for doing survival analysis. It makes use of the power and the wide variety of packages provided by R language to compute and plot the results. The ANN based model provides the user with a straightforward tool for discrete time survival analysis based on the Mani method. Although the other methods are available in commercial software like Stata or SAS, OSA stands out allowing users to conduct their studies without the need of learning complex command line syntax or complicated interfaces. Furthermore, it makes the graphical results available for download in PNG, PDF or EPS. As any web application, it can be used in every device with internet access, which permits researches to carry on with their work almost anywhere. This way, this open access application, which takes advantage of the computational power of the 27-node cluster, may be employed in helping in the field of survival analysis globally at no cost to its users. Future work includes Cox proportional-hazards for time dependent variables and new machine learning-based methods for survival analysis.
